# The effect of percussion massage therapy on the recovery of delayed onset muscle soreness in physically active young men—a randomized controlled trial

**DOI:** 10.3389/fpubh.2025.1561970

**Published:** 2025-03-26

**Authors:** Haiwei Li, Liang Luo, Jing Zhang, Peipei Cheng, Qiang Wu, Xinping Wen

**Affiliations:** ^1^School of Physical Education, Shanxi Normal University, Taiyuan, China; ^2^Department of Physical Education, The Fifth Bridge Elementary School in Guodu Street, Xi'an, China

**Keywords:** delayed onset muscle soreness, percussion massage therapy, stretching, recovery, randomized controlled trial

## Abstract

**Background:**

Delayed onset muscle soreness (DOMS) is a common condition among physically active individuals, often resulting in reduced performance and discomfort. Although percussive massage treatment (PMT) is widely used as a recovery tool, there is limited evidence supporting its efficacy compared to traditional methods such as static stretching.

**Objective:**

To investigate the effect of PMT on recovery from DOMS in physically active young men.

**Methods:**

Thirty physically active male college students were randomized into three groups: static stretching (SS) group, a short-duration PMT (S-PMT) group, and a long-duration PMT (L-PMT) group. All participants performed squats to induce DOMS, followed by interventions of static stretching, 25 min of PMT, or 40 min of PMT, respectively. Measurements included the visual analog scale (VAS) pain score, knee joint range of motion (ROM), countermovement jump (CMJ), and integrated electromyography (iEMG). These were measured at baseline (P0), post-DOMS protocol (P1), post PMT (P2), 24 h post-intervention (P3), and 48 h post-intervention (P4). Data were analyzed using repeated-measures ANOVA or nonparametric tests, with multiple comparisons conducted at a significance level of *p* < 0.05.

**Results:**

Compared with the SS and S-PMT group, the L-PMT group at P4 demonstrated significantly greater jump height (SS group: *p* < 0.001, d = 8.691; S-PMT group: *p* = 0.006, d = 4.37), peak ground reaction force (SS group: *p* < 0.001, d = 19.174; S-PMT group: *p* < 0.001, d = 14.334), and propulsion impulse (SS group: p < 0.001, d = 8.302; S-PMT group: *p* = 0.003, d = 4.517) during the CMJ propulsion phase. Additionally, the normalized iEMGs of the three muscles in the L-PMT group were significantly lower than those in the S-PMT (VM: *p* < 0.001, d = −5.692; RF: *p* < 0.001, d = −8.222; VL: *p* < 0.001, d = −10) and SS groups at P4 (VM: *p* < 0.001, d = −12; RF: *p* < 0.001, d = −11.384; VL: *p* < 0.001, d = −15). At P4, the L-PMT group exhibited significantly lower VAS scores than the SS group (*p* = 0.003, d = −1.53), as well as significantly greater knee joint ROM compared to the SS group (*p* = 0.012,d = 4.77).

**Conclusion:**

PMT was more effective than static stretching for DOMS recovery. Furthermore, two 40-min PMT sessions provided greater benefits than two 25-min sessions for treating DOMS. These findings suggest that PMT can be a valuable tool for physically active individuals seeking to enhance recovery and maintain performance.

**Clinical trial registration:**

The study was registered on ClinicalTrials.gov on September 21, 2024, with the identifier number NCT06612502.

## Introduction

Delayed onset muscle soreness (DOMS) is a common condition that typically develops within 12 to 24 h after exercise, peaks at 24 to 72 h, and usually resolves within 7 days (1). The characteristic symptoms of DOMS include reduced in strength, limited range of motion (ROM), muscle pain, stiffness, swelling, and impaired joint function (2). The precise mechanism of DOMS remains unclear, however, several hypotheses have been proposed, such as inflammation, microtrauma to muscle fibers, and oxidative stress (1, 3). There is also a general consensus that DOMS is caused by eccentric contractions or unfamiliar forms of exercise (4). Currently, the primary therapeutic modalities for DOMS include acupuncture, massage, thermal therapy, compression therapy, and nutritional interventions (5). As a physiotherapeutic intervention, massage is widely used for DOMS recovery (6). One systematic review and meta-analysis suggests that massage therapy after strenuous exercise can effectively reduce DOMS soreness ratings, improve muscle strength, and lower serum creatine kinase levels (7).One recovery tool that has recently gained prominence is the percussive massage treatment (PMT) device, commonly known as a percussive massage gun (8). The handheld PMT device is a popular recovery tool among both professional and recreational athletes (9). For instance, during the 18th Asian Games, athletes frequently used PMT devices (10). Additionally, in a regular training week, 15–25% of competitive triathletes employ these devices (11).

Researchers have conducted numerous studies on the application of the device, particularly focusing on its use in pre- and post-exercise contexts. Previous studies have shown that 2–5 min of PMT applied to the calf muscles or hamstrings can increase maximum ROM by reducing tissue stiffness (12, 13), potentially lowering the risk of injury (14). Another study used a 60-s PMT intervention to temporarily reduce Achilles tendon tissue stiffness; However, some evidence suggests that PMT may slightly impair subsequent explosive performance (15). Additionally, PMT may improve performance and balance when used before exercise (16).

The effects of PMT applied after exercise-induced fatigue or DOMS have been investigated in several studies. In a study by Alonso-Calvete et al., an 8-min session of PMT did not improve recovery in lifeguards after a 100-meter water rescue, as measured by blood lactate levels and perceived fatigue (17).Similarly, Menek et al. applied PMT for 5 min following intense calf exercise and found that PMT had minimal effect on ankle ROM, calf circumference, isometric strength, or calf endurance (16). Additionally, a single 10-min session of PMT or foam rolling was not superior to passive rest in alleviating DOMS symptoms in recreational athletes (18). Notably, PMT in these studies was limited to a single session lasting less than 10 min. In contrast, prolonged recovery interventions, such as extended massage or compression therapy, have been shown to enhance blood flow, reduce inflammation, and accelerate tissue repair (19).

Given these limitations, scholars have proposed that future studies investigating the effects of PMT on DOMS recovery should utilize longer durations and multiple sessions (18). A comprehensive understanding of the effects of PMT on DOMS recovery can lead to the optimization of treatment protocols, ultimately improving athletic performance and reducing recovery times.

Therefore, the objective of this study was to examine the impact of varying PMT durations on DOMS recovery of in physically active young men. We hypothesized that (1) PMT would be more effective than static stretching in alleviating DOMS symptoms, and (2) longer PMT sessions (40 min) would provide greater recovery benefits compared to shorter sessions (25 min).

## Materials and methods

### Ethics

The study was approval by the Science and Technology Ethics Committee of Shanxi Normal University (Approval No. 20240803), and all participants provided written informed consent. The study was registered on Clinical Trials.gov on September 21, 2024 (Identifier: NCT06612502).

### Participants

The sample size was calculated using G*Power (version 3.1.9.7; Kiel University, Germany). Based on an assumed medium effect size (*f* = 0.25) (20), three independent groups, five repeated measurements, a type I error rate of 5%, and a statistical power of 80%, a minimum of 27 participants was required for the study. To ensure sufficient statistical power, the sample size was increased by 10%, resulting in a final sample size of 30 participants.

The participants were recruited between July 1, 2024, and July 15, 2024. The recruitment process was overseen by one of the authors (LL). The inclusion criteria were as follows: (1) Male college students aged between 19 to 23 years. (2) Engagement in regular physical exercise (≥three times per week). (3) Absence of exercise contraindications, as confirmed by a physician’s health certificate or physical examination report. (4) Commitment to adhering to the experimental protocol throughout the study and refraining from any unplanned experimental interventions.

**Figure 1:**
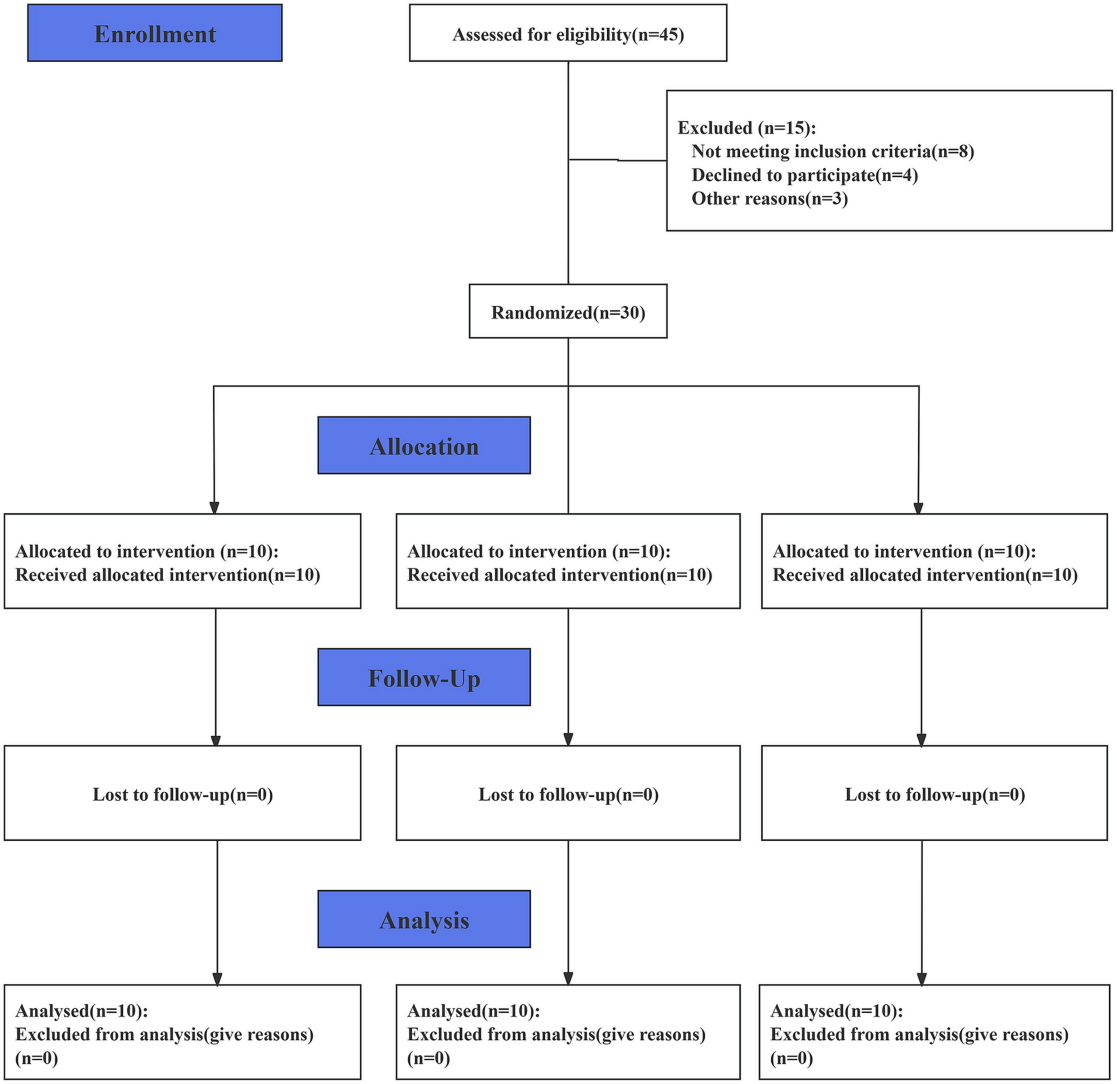
Flow chart of study participation and follow-up.

This study utilized a three-arm design, with participants randomly assigned to one of three study arms using a random number generator. The three arms were as follows: the static stretching (SS) group, the short-duration PMT (S-PMT) group (25 min of PMT treatment), and the long-duration PMT (L-PMT) group (40 min of PMT treatment). Participants were allocated to the three arms in a 1:1:1 ratio. The study was conducted in accordance with the Consolidated Standards of Reporting Trials (CONSORT) Statement. (See S1 CONSORT Checklist). After data analysis was completed, an independent consultant was responsible for the randomization and data decoding. (Please refer to Figure 1.) The participants were instructed to maintain their usual hydration, sleep, and nutritional habits throughout the study period to minimize the influence of confounding factors.

### Procedures

All participants were required to attend four testing sessions, each scheduled at the same time and in the same laboratory. The sessions were organized as follows: (1) Orientation and baseline testing prior to the experiment; (2) pretest (P0), DOMS protocol, posttest 1 (P1), interventions, and posttest 2 (P2); (3) posttest 24 h (P3), interventions; and (4) posttest 48 h (P4). All testing sessions were separated by 24 h, except for sessions 1 and 2, which were separated by at least 96 h to ensure participants had fully recovered from the predicted 1RM back squat test.

In session 1, the experimental protocol and procedures were distributed to the participants. Participants voluntarily signed an informed consent form. The following measurements were taken and recorded: height, weight, body mass index (BMI), and predicted 1RM back squat. All participants were provided with an overview of the DOMS protocol and outcome measurements. Participants in the SS group were additionally instructed to perform the SS protocol to ensure consistency in stretching exercise techniques.

In session 2, upon arrival at the laboratory, participants first completed a 10-min warm-up, followed by the initial test (P0). The sequence of all tests (P0-P5) was identical, and included the following: a visual analog scale (VAS) for perceived lower limb pain, knee joint ROM, countermovement jump (CMJ), and surface electromyography (EMG), which was performed simultaneously with the CMJ test. ROM and surface EMG measurements were conducted on the dominant leg. After the initial test, participants were given an adequate rest period. They then proceeded with the DOMS protocol, which involved performing 10 × 7 back squats under supervision. The second test (P1) was conducted immediately after the DOMS protocol. Following this, the SS group performed static stretching, while the S-PMT and L-PMT groups underwent PMT. The third test (P2) was performed after the intervention.

**Figure 2:**
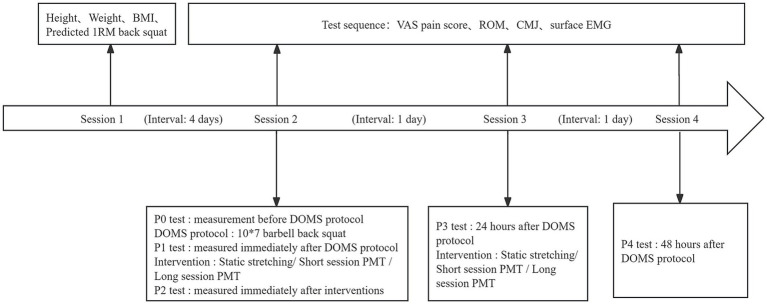
Flow chart of the experimental procedures.

In sessions 3 and 4, tests were conducted at 24 and 48 h post-DOMS protocol, respectively. The procedure and sequence of the tests were consistent with those used in the previous sessions. Following the tests in session 3, the groups underwent their corresponding recovery interventions. The outcome tests were designated P3 and P4. The experimental procedure is illustrated in Figure 2.

### DOMS protocol

The participants in this study were not professional athletes; therefore, Brzycki’s equations was used to estimate the one-repetition maximum (1RM) for the back squat test (21). The test was conducted in session 1, prior to the implementation of the DOMS protocol. The following formula was used to predict 1RM based on repetitions to fatigue with weights ranging from 45 to 310 pounds:


$$\mathrm{Predicted 1RM Back Squat}=\mathrm{Weight lifted}/\left(1.0278–0.0278\times r\right)$$

Where r represents the number of repetitions.

Each participant was allowed to warm up thoroughly prior to testing. Based on the participant’s training history, strength and training coaches assisted in selecting the test weight, recorded the weight and number of repetitions, and estimated the 1RM back squat weight. To induce DOMS, participants performed 10 sets of 7 repetitions of back squats at 60% of their 1RM, with a two-minute rest interval between sets, in accordance with established DOMS protocols (22–24). Since eccentric contraction is a crucial factor in DOMS development, the back squats were performed at a specific tempo (25). The tempo consisted of a four-second phase: a one-second eccentric contraction, a one-second pause at the bottom, a one-second concentric contraction, and a one-second pause at the top of the lift. The investigator used an interval timer to regulate the tempo and provided instructions on the lifting phase. The DOMS protocol was completed under the investigator supervision, and all the participants successfully completed the protocol during session 2. Furthermore, DOMS was confirmed through self-reported pain scores (VAS) and objective measures such as reduced knee joint ROM and jump performance.

### Interventions

#### SS group

The static stretching protocol consisted of eight exercises, each performed for 30 s per bilateral muscle group (except for the sitting toe touch and butterfly stretch), targeting the hip, knee, and ankle. Participants were instructed to perform the following sequence of stretches: forward lunge, supine knee flexion, lateral quadriceps stretch, sitting toe touch, semi straddle, straddle, butterfly stretch, and a straight knee ankle extensor wall stretch (26). Each participants completed all stretches in sequence before moving on to the next. The stretches were maintained until mild discomfort was left, but not to the point of pain.

### S-PMT and L-PMT groups

**Figure 3:**
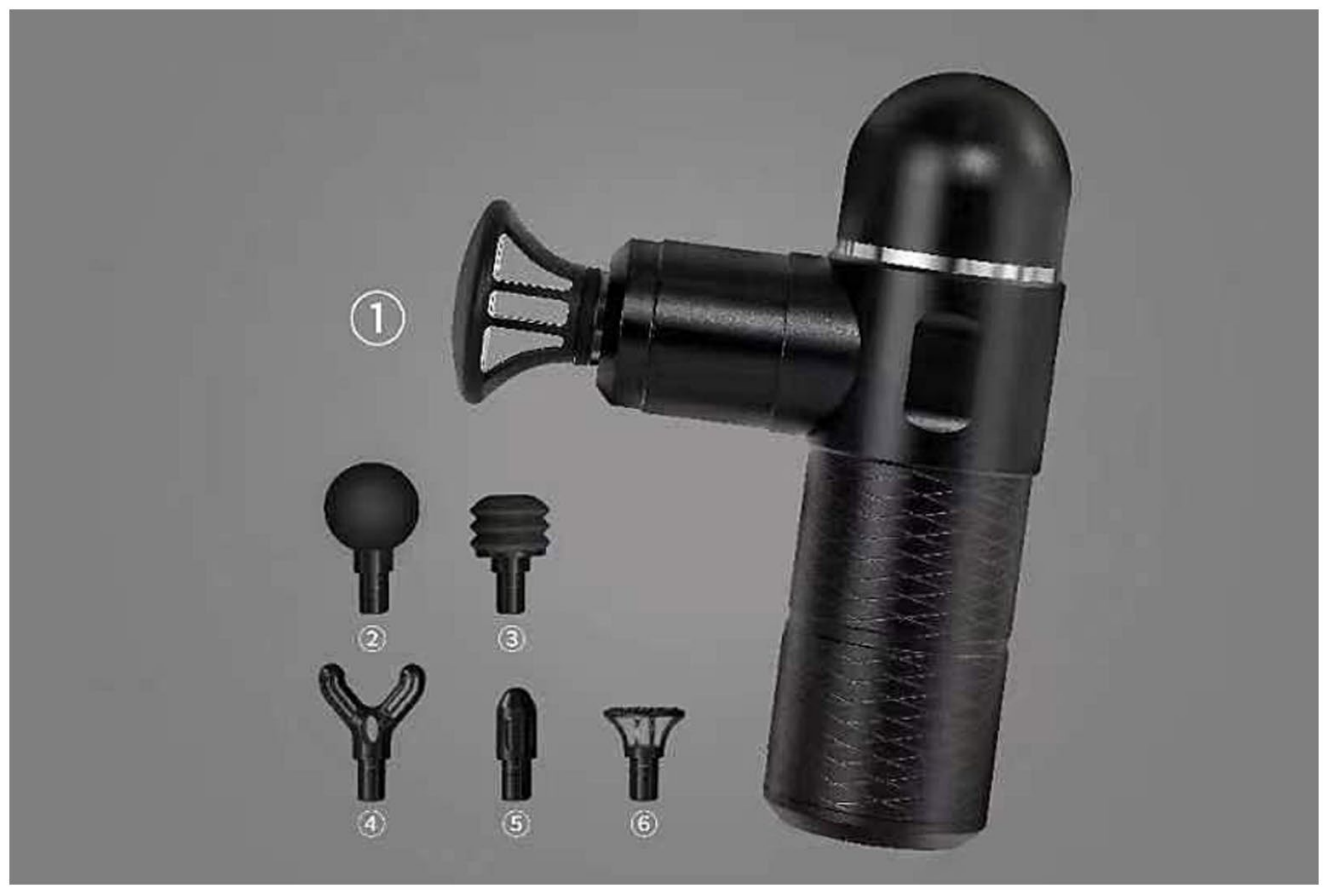
OUTSO 06 fascia gun device.

Two physiotherapists performed PMT using an OUTSO 06® fascia gun device (Jinhua Lingding Sporting Goods Co. Ltd., Zhejiang, China; see Figure 3) equipped with a 5 cm diameter soft attachment head (head ①). Previous studies have indicated that the frequency of vibration training used to relieve muscle pain typically ranges from 50 to 200 Hz (27). A vibration frequency of 53 Hz has been frequently selected for PMT interventions (12, 17). Therefore, 53 Hz was selected as the vibration frequency in this study. The device exhibited an amplitude of 6 mm and a torque of 33 pounds (12).

PMT was performed directly after tests P1 and P3. We chose these two times points for the following reasons: First, although DOMS symptoms are not particularly noticeable after the DOMS protocol, early massage aids in lactic acid clearance and tissue healing (19); second, DOMS symptoms typically peak 24–48 h after exercise (28).

In this study, the selection and sequence of muscle groups for PMT were determined according to established methods (22). Throughout the PMT sessions, physiotherapists carefully maintain a consistent level of moderate pressure on the skin and follow the direction of the muscle fibers as outlined in a previous protocol (12). The targeted muscles, postures, PMT application details, and recovery durations for the S-PMT and L-PMT groups are presented in Table 1.

**Table 1:** The muscles, posture, application of PMT, and duration of recovery in the two groups.

Muscles	Positions	Methods	Duration	
			S-PMT	L-PMT
Quadriceps	Prone	The PMT device was initiated at the most medial aspect of the treated muscles and then moved in a linear trajectory from the distal to the proximal region and back to the distal region within a time frame of 10 to 20 s. At the distal end of the muscles, the investigator then proceeded to move the PMT device laterally and subsequently moved it in a longitudinal direction from the distal to the proximal region and back to the distal region. This process was repeated until the full session time was reached.	2.5 min per site, 25 min in total	4 min per site, 40 min in total
Adductors	Prone with the hip flexed and externally rotated			
Hamstrings	Supine			
Iliotibial Band	Side-lying			
Gluteals	Supine			

### Outcome measurements

#### Muscle pain

The intensity of muscle pain was quantified using the VAS. The VAS consists of a 10-cm straight line with one end labeled “no pain” (score of 0) and the opposite end labeled “intolerable pain” (score of 10) (29). Participants were required to provide a rating of their perceived lower-limb muscle soreness for each leg on a scale of 0 to 10 (30). Two measurements were taken using the VAS, and the average score was calculated.

#### Knee joint ROM

A modified kneeling lunge was used to assess knee joint ROM (31). The procedure was as follows: the participants left leg was bent at the knee, with the thigh parallel to the ground and the left lower leg perpendicular to the ground. The right leg was also bent at the knee, while the upper body was maintained in an upright position. The participant adjusted their position to stretch the right hip to the point of discomfort. The angle of the right hip stretch was measured and used as a criterion for subsequent measurement of the ROM. The investigator then bent the subject’s right knee until the point of discomfort was reached. At this point, the angle between the thigh and calf was recorded using a goniometer with the following anatomical landmarks: the lateral malleolus, the lateral epicondyle, and the center of the vastus lateralis. A larger angle indicates a smaller ROM of the knee joint.

#### CMJ

The CMJ was performed following established methodology as described in the literature (32). CMJ variables were evaluated using a BTS P-6000 three-dimensional force platform (BTS Bioengineering, Milan, Italy), with a sampling frequency of 1,000 Hz. Each participant performed three trials, and the highest vertical jump height was selected for analysis. The variables measured included jump height, peak ground reaction force (GRF) during the propulsion phase, and the propulsion impulse. Jump height was calculated based on the time spent in the air (33). Peak GRF was derived from the force–time curve, and the calculation of the propulsion impulse was based on the previous literature (34, 35).

#### Surface EMG

In this study, wireless sEMG (Noraxon USA Inc., Scottsdale, AZ, USA) was used to collect integrated EMG (iEMG) signals from the vastus medialis (VM), rectus femoris (RF), and vastus lateralis (VL) during the CMJ on the dominant leg, with a sampling frequency of 1,000 Hz. The skin surface was prepared using abrasion and alcohol swabs. Disposable Ag–AgCl circular surface electrodes (4 mm diameter) were filled with electrode jelly and attached 2.5 cm apart on each muscle. Raw EMG data were processed using Noraxon TeleMyo software (Noraxon USA Inc., Scottsdale, AZ, USA) for rectification, smoothing, and filtering. To eliminate the effect of time phase, the signal was partitioned in 10-ms windows to find for each muscle and each participant its maximal activation over CMJ (iEMGmax). The iEMGmax value was normalized to 100%. The iEMG during the CMJ duration was then divided by the time (iEMG/T) and expressed as a percentage of iEMGmax (36).

### Statistical analyses

The study data were analyzed using SAS 6.0 software (SAS Institute Inc., Cary, NC, USA). Data normality was assessed using the Shapiro–Wilk test with a significance level of 0.05. Additionally, QQ plots and histograms were generated to visually confirm normality. Sphericity was evaluated using Mauchly test, with a significance level of 0.05.

Results were expressed as means ± standard deviation (SD) or as medians (with ranges). A two-way repeated-measures ANOVA was conducted to examine the main effects of group and time, as well as the group × time interaction effect for the variables knee joint ROM, CMJ, and iEMG, as these variables met the assumptions of normality and sphericity. For the VAS, which did not satisfy the assumption of normality, the Kruskal-Wallis test (for between-group comparisons) and the Friedman test (for within-group time effects) were employed for analysis. Post-hoc analyses were performed using the Bonferroni correction to adjust for multiple comparisons. Effect sizes (ES) were reported as eta squared (η^2^) for ANOVA and Kruskal-Wallis test, Kendall’s W for the Friedman test and Cohen’s d for group comparisons (37). The significance level was set to *p* < 0.05.

## Results

### Participants

A total of 30 male college students participated in the study, and none withdrew. Recruitment, data collection and analysis took place between July 2024 and August 2024. Detailed information regarding the study flow is illustrated in the corresponding flow chart (Figure 1). Table 2 presents the baseline characteristics of the three groups, which showed no significantly differences.

**Table 2:** Characteristics of the three groups.

Subjects	SS (*n* = 10)	S-PMT (*n* = 10)	L-PMT (*n* = 10)
Age, y	21.22 ± 1.66	21.55 ± 2.21	21.77 ± 2.16
Height, cm	171.46 ± 4.44	171.46 ± 4.44	173.52 ± 5.58
weight, kg	66.87 ± 7.46	67.83 ± 10.88	67.06 ± 11.95
BMI, kg/m^2^	20.90 ± 2.14	22.23 ± 2.14	22.08 ± 2.95
1-RM squat, kg	46.73 ± 9.18	47.09 ± 5.95	48.64 ± 4.88

### VAS

The medians (with ranges) for the VAS score are presented in Table 3. A Friedman test was conducted to examine the differences in VAS across five repeated measures in the three groups. The results indicated statistically significant difference between the five repeated measures in all three groups (SS: *p* < 0.001, Kendall’s W = 0.762; S-PMT: *p* < 0.001, Kendall’s W = 0.786; L-PMT: *p* < 0.001, Kendall’s W = 0.910). Post-hoc pairwise comparisons with Bonferroni correction revealed that the VAS scores were highest at P3 in all three groups. However, the VAS scores in the L-PMT group at P4 was significantly lower than those at P3 (*p* = 0.001, d = 4.01).

**Table 3:** VAS scores for three groups at different time points.

Time	SS (*n* = 10)	S-PMT (*n* = 10)	L-PMT (*n* = 10)
P0	0 (0)	0 (0)	0 (0)
P1	2 (1.25)	1.5 (1.00)^A^	2 (0)^AA^
P2	1 (0.25)	1 (0.25)	1 (0)
P3	3.5 (1.50)^AABC^	3 (0.25)^AAC^	3 (1.00)^AA^
P4	2.5 (1.50)^A^	1.5 (1.00)^A^	0 (1.00)^BDD▲▲^

^A and AA indicate p < 0.05 and p < 0.01 compared with P0, respectively; B and BB indicate p < 0.05 and p < 0.01 compared with P1, respectively; C and CC indicate p < 0.05 and p < 0.01 compared with P2, respectively; D and DD indicate p < 0.05 and p < 0.01 compared with P3, respectively. ▲ and ▲▲ indicate p < 0.05 and p < 0.01, respectively, compared with the SS group.^

A Kruskal-Wallis test was conducted to examine the differences in VAS among the three groups. At P4, the results indicated a statistically significant difference between the groups (*p* = 0.003, η^2^ = 0.397). Post-hoc pairwise comparisons with Bonferroni correction revealed that the VAS scores in the L-PMT group was significantly lower than those in the SS (*p* = 0.003, d = 1.53).

### Knee joint ROM

The means and SDs for the knee joint ROMs are presented in Table 4. Significant main effects of time (*p* < 0.001, η^2^ = 0.670) and a significant interaction (*p* < 0.015, η^2^ = 1.99) were observed. *Post hoc* comparison of ROM at different time points within each group revealed a significant increase at P1 and P2 in all groups (both *p* < 0.05) and a significant decrease at P3 and P4 in the SS group compared to P0 (both p < 0.01). Post hoc analysis of the three groups at the same time point revealed that the ROM was significantly greater in the L-PMT group than in Group SS at both P3 (*p* = 0.006, d = −5.34) and P4 (*p* = 0.012, d = −4.77).

**Table 4:** Knee joint ROM for three groups at different time points (°).

Time	SS (*n* = 10)	S-PMT (*n* = 10)	L-PMT (*n* = 10)
P0	55.10 ± 0.96	55.89 ± 1.06	56.32 ± 1.32
P1	51.78 ± 1.47^A^	52.59 ± 1.22^AA^	52.99 ± 1.28^AA^
P2	51.86 ± 1.44^A^	51.37 ± 1.01^AAB^	49.69 ± 0.92^AABB^
P3	67.25 ± 1.95^AABBCC^	62.49 ± 2.57^BCC^	59.08 ± 0.93^BBCC▲▲^
P4	63.31 ± 1.30^AABBCC^	61.42 ± 2.20^BCC^	56.92 ± 1.38^BBCC▲^

^A and AA indicate p < 0.05 and p < 0.01 compared with P0, respectively; B and BB indicate p < 0.05 and p < 0.01 compared with P1, respectively; C and CC indicate p < 0.05 and p < 0.01 compared with P2; respectively. ▲ and ▲▲ indicate p < 0.05 and p < 0.01, respectively, compared with the SS group.^

### CMJ

The means and SDs for jump height, peak GRF, and propulsion impulse are presented in Tables 5. The main effect of group on peak GRF was significant (*p* < 0.001, η^2^ = 0.466). Additionally, significant main effects of time (Jump heights: *p* < 0.001, η^2^ = 0.699; Peak GRFs: *p* < 0.001, η^2^ = 0.712; Propulsion impulses: *p* < 0.001, η^2^ = 0.765) and significant time × group interaction (Jump heights: *p* < 0.001, η^2^ = 0.291; Peak GRFs: *p* < 0.001, η^2^ = 0.375; Propulsion impulses: *p* < 0.001, η^2^ = 0.436) were observed for all three variables. Comparisons of different time points within the three groups revealed that, compared to P0, all three CMJ-related variables significantly decreased at P1, P2, and P3 (all *p* < 0.05). Comparison of the three groups at the same time point revealed that all three variables were significantly greater in the L-PMT than in Group SS (Jump heights: *p* < 0.001, d = 8.691; Peak GRFs: *p* < 0.001, d = 19.174; Propulsion impulses: *p* < 0.001, d = 8.302) and Group S-PMT (Jump heights: *p* = 0.006, d = 4.37; Peak GRFs: *p* < 0.001, d = 14.334; Propulsion impulses: *p* = 0.003, d = 4.517) at P4.

**Table 5:** CMJ of the three groups at different time points.

CMJ	Time	SS (*n* = 10)	S-PMT (*n* = 10)	L-PMT (*n* = 10)
Jump heights (cm)	P0	31.12 ± 0.43	30.48 ± 0.51	30.52 ± 0.47
	P1	28.02 ± 0.56^AA^	28.07 ± 0.31^AA^	27.93 ± 0.27^AA^
	P2	27.86 ± 0.64^AA^	28.00 ± 0.29^AA^	27.79 ± 0.28^AA^
	P3	28.06 ± 0.34^AA^	27.29 ± 0.35^AABBC^	27.00 ± 0.37^AABBCC▲^
	P4	28.03 ± 0.30^AA^	29.27 ± 0.28^BBCDD▲▲^	30.43 ± 0.25^BBCCDD▲▲¥¥^
Peak GRFs (N)	P0	1391.24 ± 4.40	1394.57 ± 5.40	1393.47 ± 5.57
	P1	1338.15 ± 6.34^AA^	1339.58 ± 8.50^AA^	1337.58 ± 16.83^A^
	P2	1331.78 ± 11.16^AA^	1335.13 ± 8.75^AA^	1341.66 ± 7.57^AA^
	P3	1267.25 ± 5.41^AABBCC^	1282.62 ± 13.64^AABBCC^	1302.28 ± 10.82^AAC▲^
	P4	1272.14 ± 6.33^AABBCC^	1295.87 ± 6.77^AABBC▲^	1380.34 ± 4.86^BCCDD▲▲¥¥^
Propulsion impulses (N.s)	P0	188.21 ± 2.81	188.41 ± 1.92	186.17 ± 2.34
	P1	175.14 ± 2.28^AA^	173.67 ± 2.42^AA^	171.43 ± 2.75^AA^
	P2	173.73 ± 1.20^AA^	173.08 ± 2.21^AA^	172.41 ± 2.40^AA^
	P3	166.92 ± 2.23^AABBCC^	170.83 ± 1.73^AA^	170.26 ± 2.18^AA^
	P4	169.34 ± 1.92^AABBCDD^	175.97 ± 2.28^AA▲^	185.70 ± 2.02^BBCCDD▲▲¥¥^

^A and AA indicate p < 0.05 and p < 0.01 compared with P0, respectively; B and BB indicate p < 0.05 and p < 0.01 compared with P1, respectively; C and CC indicate p < 0.05 and p < 0.01 compared with P2, respectively; D and DD indicate p < 0.05 and p < 0.01 compared with P3, respectively. ▲ and ▲▲ indicate p < 0.05 and p < 0.01, respectively, compared with the SS group. ¥ and ¥¥ indicate p < 0.05 and p < 0.01, respectively, compared with the S-PMT group.^

### Normalized iEMG

The means and SDs of the normalized iEMG are presented in Tables 6. Significant main effect of group was observed for the normalized iEMG of the rectus femoris (*p* < 0.001, η^2^ = 0.660). Additionally, the main effects of time (VM: *p* < 0.001, η^2^ = 0.634; RF: *p* < 0.001, η^2^ = 0.736; VL: *p* < 0.001, η^2^ = 0.787) and the time × group interaction (VM: *p* < 0.001, η^2^ = 0.257; RF: *p* < 0.001, η^2^ = 0.363; VL: *p* < 0.001, η^2^ = 0.583) on the normalized iEMG of the three muscles were statistically significant. Comparison of different time points within the three groups revealed that the normalized iEMGs of the three muscles were significantly higher at P1, P2, and P3 compared to P0 (*p* < 0.01 for all). Furthermore, the normalized iEMGs were also significantly higher in the SS and S-PMT groups at P4 (*p* < 0.01 for all). When comparing the three groups at the same time point, the normalized iEMGs of the three muscles in the L-PMT group were significantly lower than those in the S-PMT (VM: *p* < 0.001, d = −5.692; RF: *p* < 0.001, d = −8.222; VL: *p* < 0.001, d = −10) and SS groups at P4 (VM: *p* < 0.001, d = −12; RF: *p* < 0.001, d = −11.384; VL: *p* < 0.001, d = −15).

**Table 6:** Normalized iEMG of the three muscles for the three groups at different time points (%).

Quadriceps Femoris	Time	SS (*n* = 10)	S-PMT (*n* = 10)	L-PMT (*n* = 10)
VM	P0	0.66 ± 0.02	0.68 ± 0.02	0.68 ± 0.02
	P1	0.72 ± 0.02^AA^	0.73 ± 0.02^AA^	0.72 ± 0.02^AA^
	P2	0.75 ± 0.02^AAB^	0.78 ± 0.02^AABB^	0.74 ± 0.02^AA^
	P3	0.82 ± 0.01^AABBCC^	0.82 ± 0.01^AABBCC^	0.79 ± 0.02^AABCC^
	P4	0.82 ± 0.01^AABBCC^	0.79 ± 0.02^AAB^	0.70 ± 0.01^BDD▲▲¥¥^
RF	P0	0.73 ± 0.02	0.72 ± 0.02	0.70 ± 0.01
	P1	0.80 ± 0.01^AA^	0.79 ± 0.01^AA^	0.77 ± 0.01^AA^
	P2	0.84 ± 0.01^AAB^	0.83 ± 0.01^AABB^	0.81 ± 0.01^AABB^
	P3	0.93 ± 0.01^AABBCC^	0.89 ± 0.01^AABBCC▲^	0.85 ± 0.01^AABBC▲▲¥^
	P4	0.90 ± 0.02^AABBCC^	0.85 ± 0.02^AA▲^	0.72 ± 0.01^BCCDD▲▲¥¥^
VL	P0	0.62 ± 0.01	0.62 ± 0.01	0.61 ± 0.01
	P1	0.70 ± 0.01^AA^	0.71 ± 0.01^AA^	0.73 ± 0.01^AA▲^
	P2	0.70 ± 0.01^AA^	0.71 ± 0.01^AA^	0.73 ± 0.01^AA▲^
	P3	0.75 ± 0.01^AABCC^	0.76 ± 0.01^AABBCC^	0.75 ± 0.01^AA^
	P4	0.78 ± 0.01^AABBCC^	0.73 ± 0.01^AABCDD▲▲^	0.63 ± 0.01^BBCCDD▲▲¥¥^

^A and AA indicate p < 0.05 and p < 0.01 compared with P0, respectively; B and BB indicate p < 0.05 and p < 0.01 compared with P1, respectively; C and CC indicate p < 0.05 and p < 0.01 compared with P2, respectively; D and DD indicate p < 0.05 and p < 0.01 compared with P3, respectively. ▲ and ▲▲ indicate p < 0.05 and p < 0.01, respectively, compared with the SS group. ¥ and ¥¥ indicate p < 0.05 and p < 0.01, respectively, compared with the S-PMT group.^

## Discussion

The purpose of our study was to evaluate the effects of PMT on recovery from DOMS in physically active male college students. The results demonstrated that PMT significantly alleviated pain, accelerated the recovery of lower limb flexibility and strength, and improved the electrophysiological properties of the lower limb muscles. Furthermore, PMT achieved superior recovery outcomes compared to static stretching. Additionally, the study revealed that the recuperative benefits of 40 min of PMT surpassed those of 25 min.

Studies have consistently used VAS to assess DOMS-related pain, as it correlates well with the degree of muscle damage and inflammation (25). Peak muscle pain in all three groups occurred 24 h after the DOMS protocol. The VAS scores in the L-PMT group were lower than those in the SS and S-PMT groups at P4. Romero-Miralda et al. (38) induced muscle damage through eccentric exercise and compared intervention using a vibrating foam roller (18 Hz) and a non-vibrating foam roller. The study concluded that the vibrating foam roller significantly alleviated muscle soreness 48 h post-injury. Similarly, Lau et al. (27) applied PMT therapy at a frequency of 65 Hz and an amplitude of 1 mm for 30 min daily over 5 days. This intervention reduced VAS scores by 18 to 30% and accelerated the alleviated of DOMS symptoms in 15 young men following eccentric exercise. These findings align with the results of the present study. In contrast, Dabbs et al. (39) found that whole-body vibration training at 30 Hz with an amplitude of 2–4 mm was ineffective in alleviating DOMS induced by high-intensity exercise in young women. This finding is inconsistent with the results of the present study and may be attributed to the disparate treatment methods employed for whole-body and local vibration. Currently, the mechanism by which PMT reduces DOMS-related pain remains unclear. Some scholars propose that PMT stimulates non-nociceptive input from large-diameter fibers, enhancing spinal inhibition of nociceptive input (40, 41). Others suggest that PMT improves blood circulation, increases oxygen saturation, and facilitates the elimination of pain-causing substances, thereby alleviating pain in DOMS (38).

Measuring knee ROM is therefore a useful objective indicator of DOMS severity and recovery, as improvements in ROM often parallel reductions in muscle soreness and inflammation (42). A notable increase in the ROM of the knee joints was observed in all three groups at P1 and P2. This improvement may be attributed to reduced muscle viscosity resulting from elevated muscle temperature post-exercise (43). The ROM of the knee joints was at its lowest across all groups at P3. By P4, the ROM began to recover in all groups, with the L-PMT group demonstrating superior recovery compared to the SS group. A study evaluating the short-duration effects of a wearable vibration device following intense eccentric exercises of the elbow flexors found that vibration therapy at a frequency of 120 Hz and an amplitude of 1.2 mm, applied for 15 min immediately post-exercise, significantly improved ROM at 24 h, 48 h, and 72 h (all *p* < 0.05) (44). Similarly, Lau applied 30 min of vibration therapy (frequency: 65 Hz, amplitude 1 mm) after eccentric exercise of the elbow flexors and observed significantly faster ROM recovery at 3–7 days post-exercise (*p* = 0.01) (27). These findings align with those of the present study. PMT has been proven to elevate muscle temperature and stimulate blood flow (45). Additionally, it generates pressure and friction at the of muscle-fascia interface, which may reduce movement resistance (12).

Vertical jump performance is a functional measure of lower limb power and neuromuscular efficiency. This relationship is well-documented, as DOMS-induced muscle damage negatively affects explosive movements like jumping (46). Jump height, peak GRF and propulsive impulse were significantly higher in the L-PMT and S-PMT groups compared to the SS group at P4 (all *p* < 0.05). Furthermore, a comparison between the L-PMT and S-PMT groups showed that all three variables were significantly higher in the former (all *p* < 0.01) at P4. These result suggests that PMT can promote recovery of muscle strength after DOMS, and that a 40-min session is more effective than a 25-min session. In a related study, scholars used a PMT device to relax the gastrocnemius for 5 min and found that this intervention did not enhance the muscle’s strength. However, it is important to note that the study examined the effects of PMT on the gastrocnemius in its normal state, not after the onset of DOMS (12). Timon, R. et al. observed that a single session of whole-body vibration training intervention (frequency: 12 Hz, amplitude: 4 mm, duration:3 min) did not promote the recovery of quadriceps strength after DOMS (47). Similarly, Ansari, N. and other scholars found that a single session of whole-body vibration training intervention (frequency: 30 Hz, amplitude: 4 mm, duration:2 min) did not promote the recovery of isometric peak moment or single-leg jump distance in quadriceps after fatigue (48). The results of the two studies were inconsistent with the results of the present study, probably because whole-body vibration training was used, and the duration of vibration was short and the frequency of vibration was low. The potential mechanisms by which PMT facilitates the restoration of lower extremity muscle strength can be categorized into two main pathways: Initially, PMT can stimulate the recruitment of a greater number of type I and type II muscle fibers, thereby enhancing muscle contractility (49). Second, PMT promotes muscle vasodilation, increases oxygen delivery, and enhances blood volume. Thereby facilitating the recovery of strength qualities (38).

DOMS often results in altered muscle recruitment strategies and reduced motor unit activation, which can be quantified using EMG (23). The normalized iEMG of the target muscles in all three groups increased significantly from P1 to P3. At P4, the S-PMT group (RF and VL) and the L-PMT group (RF, VL, and VM) exhibited significantly lower normalized iEMG compared to the SS group. Notably, the normalized iEMG in the L-PMT group had returned to normal, outperforming the S-PMT group at P4. Studies have shown that the elbow flexor and quadriceps muscles exhibited significant increase in EMG amplitude and iEMG during submaximal eccentric exercise. This phenomenon may be related to muscle fiber damage caused by eccentric exercise, which requires the recruitment of additional motor units and enhances muscle fiber discharge synchronization during contraction (50, 51). These findings are consistent with the results of the present study. In another study, upper limb wrist flexion and extension exercises were used to induced forearm fatigue, followed by an intervention using the Power Plate whole-body vibration trainer (30 Hz, 2 mm, 3 min). The study found a significant decrease in upper limb iEMG (52), which aligns with the findings of this study. To date, no clear mechanisms have been established to explain the exact effect of PMT on DOMS. One potential mechanism is that PMT may facilitate the activation of motor units by stimulating α-motor neurons and the γ-system, thereby enhancing neuromuscular electrophysiology (53). However, the causal relationship between muscle strength recovery and improvement in muscle electrophysiology requires further investigation.

## Limitations

This study has several limitations. First, the participants were limited to physically active male college students, which may restrict the generalizability of the findings to the broader population. Future studies should include diverse populations. Second, the PMT device used in this study is equipped with different types of massage heads, which may yield varying intervention effects; Future research could explore the impact of different massage heads on recovery from DOMS. Third, the vibration frequency of the device was fixed at 53 Hz, and the effects of different frequencies on DOMS recovery were not examined. Fourth, while no injuries related to the use of the PMT device were observed during this study, severe cases of rhabdomyolysis have been reported following post-exercise PMT use (54). Therefore, it is essential to strictly adhere to the device’s instructions during application.

## Conclusion

PMT can facilitate recovery from eccentric exercise-induced DOMS by reducing pain, enhancing flexibility and strength recovery, and improving neuromuscular electrophysiology. PMT is more effective than static stretching in alleviating DOMS symptoms. Additionally, two 40-min PMT sessions were significantly more effective for recovery than two 25 min sessions. These findings support the use of PMT as a practical recovery tool for active individuals, offering a viable alternative to traditional methods. Coaches and practitioners may consider incorporating PMT into recovery protocols to enhance performance and reduce discomfort.

## Data Availability

The raw data supporting the conclusions of this article will be made available by the authors, without undue reservation.
